# Deciphering the Prognostic Implications of the Components and Signatures in the Immune Microenvironment of Pancreatic Ductal Adenocarcinoma

**DOI:** 10.3389/fimmu.2021.648917

**Published:** 2021-03-10

**Authors:** Rong Tang, Xiaomeng Liu, Chen Liang, Jie Hua, Jin Xu, Wei Wang, Qingcai Meng, Jiang Liu, Bo Zhang, Xianjun Yu, Si Shi

**Affiliations:** ^1^Department of Pancreatic Surgery, Fudan University Shanghai Cancer Center, Shanghai, China; ^2^Department of Oncology, Shanghai Medical College, Fudan University, Shanghai, China; ^3^Shanghai Pancreatic Cancer Institute, Shanghai, China; ^4^Pancreatic Cancer Institute, Fudan University, Shanghai, China

**Keywords:** single cell sequencing, immune microenvironment, pancreatic cancer, prognosis, tumor immune

## Abstract

**Background:** The treatment modalities for pancreatic ductal adenocarcinoma (PDAC) are limited and unsatisfactory. Although many novel drugs targeting the tumor microenvironment, such as immune checkpoint inhibitors, have shown promising efficacy for some tumors, few of them significantly prolong the survival of patients with PDAC due to insufficient knowledge on the tumor microenvironment.

**Methods:** A single-cell RNA sequencing (scRNA-seq) dataset and seven PDAC cohorts with complete clinical and bulk sequencing data were collected for bioinformatics analysis. The relative proportions of each cell type were estimated using the gene set variation analysis (GSVA) algorithm based on the signatures identified by scRNA-seq or previous literature.

**Results:** A meta-analysis of 883 PDAC patients showed that neutrophils are associated with worse overall survival (OS) for PDAC, while CD8+ T cells, CD4+ T cells, and B cells are related to prolonged OS for PDAC, with marginal statistical significance. Seventeen cell categories were identified by clustering analysis based on single-cell sequencing. Among them, CD8+ T cells and NKT cells were universally exhausted by expressing exhaustion-associated molecular markers. Interestingly, signatures of CD8+ T cells and NKT cells predicted prolonged OS for PDAC only in the presence of “targets” for pyroptosis and ferroptosis induction. Moreover, a specific state of T cells with overexpression of ribosome-related proteins was associated with a good prognosis. In addition, the hematopoietic stem cell (HSC)-like signature predicted prolonged OS in PDAC. Weighted gene co-expression network analysis identified 5 hub genes whose downregulation may mediate the observed survival benefits of the HSC-like signature. Moreover, trajectory analysis revealed that myeloid cells evolutionarily consisted of 7 states, and antigen-presenting molecules and complement-associated genes were lost along the pseudotime flow. Consensus clustering based on the differentially expressed genes between two states harboring the longest pseudotime span identified two PDAC groups with prognostic differences, and more infiltrated immune cells and activated immune signatures may account for the survival benefits.

**Conclusion:** This study systematically investigated the prognostic implications of the components of the PDAC tumor microenvironment by integrating single-cell sequencing and bulk sequencing, and future studies are expected to develop novel targeted agents for PDAC treatment.

## Introduction

Pancreatic ductal adenocarcinoma (PDAC) is the most aggressive gastrointestinal tumor, with a 5-year overall survival (OS) rate of ~9% (1). Unfortunately, the treatment modalities for PDAC remain limited and unsatisfactory (2, 3). Although many novel drugs targeting the tumor microenvironment, such as immune checkpoint inhibitors (ICIs), have shown promising efficacy in the clinic for some tumors (4–6), few significantly prolong the survival of patients with PDAC due to insufficient knowledge on the tumor microenvironment (7).

In fact, the crosstalk between tumor cells and stromal components could inspire many initiatives for novel treatment modalities. For example, a recent study showed that CD8+ T cells could induce ferroptosis, which is a non-apoptotic cell death mechanism, in multiple tumor cells, and this antitumor efficacy could be expanded by combination with ICIs (8). Similarly, CD8+ T cells and NKT cells could induce pyroptosis in tumors, and pyroptotic tumor cells reciprocally trigger more robust anticancer immunity (9–11). In addition, many studies have demonstrated that cancer-associated fibroblasts (CAFs) promote tumor proliferation and metastasis via the secretion of various cytokines (12, 13). These studies suggested that enhanced treatment efficacy could be achieved when targeting the crosstalk between tumor cells and stromal components.

Hence, elucidating the role of the components and signatures of the tumor microenvironment is significant and imperative. The advancement of single-cell sequencing technology has provided researchers with a high-throughput method for interpreting intratumoral heterogeneity by presenting the molecular characteristics of various cell components. However, a stringent limitation of single-cell sequencing is the difficulty of correlating the sequencing findings with patients' clinical information, such as survival expectancy. In this context, appropriate combination with the strength of single-cell and bulk sequencing results would optimize the utilization of sequencing data, given the availability of complete clinical information in bulk sequencing cohorts. Many single-cell sequencing-based studies have realized this limitation and tended to confirm their findings in traditional bulk sequencing cohorts (14, 15).

An increasing number of algorithms have been generated to estimate the percentage of intratumorally infiltrated stromal cells using transcriptome data (16–18). However, the association between infiltrated stromal cells in the tumor microenvironment and patient prognoses has not yet been well-established in PDAC, especially for some immune cells that theoretically exert antitumor functions. A major reason that potentially accounts for this phenomenon might be that each type of stromal cell is subdivisible and that different subtypes of a specific cell cluster may mediate contrary functions (19, 20). A classic example is the opposite roles of M1- and M2-polarizing macrophages—the former suppress tumor development, while the latter promote tumor progression in some kinds of tumors (21, 22).

Here, we annotated the cell clusters in PDAC using single-cell sequencing data and comprehensively analyzed their prognostic implications with bulk sequencing data. Multiple bioinformatic methods and *ex silico* experiments were used to identify the prognosis-related molecular traits and potential treatment targets of PDAC.

## Methods

### Sources of Datasets

A single-cell sequencing dataset (GSE155698) including 16 PDAC and 3 adjacent normal samples was obtained from the Gene Expression Omnibus (GEO). The bulk sequencing datasets were derived from The Cancer Genome Atlas (TCGA) (TCGA-PAAD), International Cancer Genome Consortium (ICGC) (ICGC-AU), GEO (GSE21501, GSE57495, GSE71729, and GSE85916), and ArrayExpress (E-MTAB-6134) databases. Both the transcriptome information and clinical information of each dataset were concurrently downloaded from the respective websites. The transcriptome data were transformed to the format of Log_2_[transcripts per million (TPM) + 1]. Only PDAC tissues were included in the subsequent analysis, while other histological subtypes, such as neuroendocrine tumors, acinar cell carcinoma, and intraductal papillary mucinous neoplasms, were excluded. T-exhaust and immune checkpoint blockade (ICB) resistance signatures were downloaded from the Tumor Immune Dysfunction and Exclusion (TIDE) database.

### Bioinformatics Analysis

#### Estimation of Intra-Tumoral Infiltrated Immune Cells

The fractions of six infiltrated immune cells, namely, CD8+ T cells, CD4+ T cells, B cells, macrophages, neutrophils, and dendritic cells, were estimated using Tumor IMmune Estimation Resource (TIMER) 2.0 (23). We also estimated the proportions of infiltrated immune cells using other algorithms, such as XCELL, CIBERSORT, and MCP-counter, in TIMER 2.0 (23). Univariate Cox regression was performed using the R package “survival.”

### Meta-Analysis of the Prognostic Implications of Infiltrated Immune Cells for PDAC

The hazard ratio (HR) for each infiltrated immune cell against the OS of PDAC was computed with the log-rank test. The HRs of each immune cell in different bulk sequencing-based cohorts were pooled in a fixed-effects model if no robust heterogeneity was observed (*I*^2^ < 50% and *P* > 0.05). The meta-analysis was performed using Stata 15.1, and the forest plot was depicted via GraphPad Prism 7.0.

### Processing of Single-Cell RNA Sequencing (scRNA-seq) Data

The “Seurat” package was used to perform the single-cell sequencing analysis. The batch effect of studies was removed through regularized negative binomial regression by the “Seurat” package (24). Genes detected in <3 cells were excluded, and cells with <200 total detected genes were excluded. Afterwards, we calculated the standardized variance of each gene across different cells, and only the top 2,000 variable genes were selected for subsequent analysis. Principal component analysis (PCA) was performed to identify significant dimensions with *P* < 0.05 (25). Then, the t-distributed stochastic neighbor embedding (tSNE) algorithm was applied for dimensionality reduction with the 20 initial PCs and for performing cluster classification analysis across all cells (26). Non-linear dimensional reduction was also performed with the UMAP method. Then, different cell clusters were determined and annotated by the “singleR” package according to the composition patterns of the marker genes and were then manually verified and corrected with the CellMarker database (27, 28). Given that both “singleR” and CellMarker could only classify cell clusters into basic types, we also referred to previously published scRNA-seq analyses to further classify each cell cluster into more precise subtypes (14, 15, 29–31).

### Single-Sample Gene Set Enrichment Analysis (ssGSEA)

The enrichment scores of the hallmark genes were evaluated using ssGSEA with the R package “GSVA” (32). Hallmark genes were defined as the top 50 genes with the largest fold change (FC) in each cluster. Using ssGSEA, each sample with complete bulk sequencing data and clinical information was labeled with an enrichment score of the specific hallmark gene signature. The samples were divided into two groups based on the median enrichment score. Then, a Kaplan-Meier curve was plotted to visualize the survival difference between the two groups. The log-rank test and Gehan-Breslow-Wilcoxon test were performed to verify the statistical significance of the survival difference. A *P* < 0.05 was regarded as indicative of a significant difference.

### Weighted Gene Co-expression Network (WGCNA)

We utilized the transcriptome profile of the E-MTAB-6134 cohort, which was the largest PDAC cohort with transcriptome and clinical data, to qualify and construct the co-expression network by the “WGCNA” package in R (33). Next, Pearson's correlation matrices were constructed for pairwise genes. We constructed a weighted adjacency matrix using a power function: a_mn_ = |c_mn_|^β^ (c_mn_ = Pearson's correlation between gene m and gene n; a_mn_ = adjacency between gene m and gene n). The β value emphasizes strong correlations between genes and penalizes weak correlations. After choosing the appropriate β value, the adjacency matrix was transformed into a topological overlap matrix (TOM), which measures the network connectivity of a gene defined as the sum of its adjacency with all other genes for network construction. To divide genes with similar expression patterns into gene modules, average linkage hierarchical clustering was performed according to the TOM-based dissimilarity measure with a minimum size of 50 for the gene dendrogram. Then, we further calculated the dissimilarity of module eigengenes (MEs), chose a cut line for the module dendrogram and merged some modules.

MEs were regarded as the major component in PCA for each gene module. We calculated the correlation between MEs and clinical traits or hallmark gene signatures to identify the relevant modules. Gene significance (GS) was defined as the log10 transformation of the *P* value in the linear regression between the gene expression level and clinical data. In addition, module significance (MS) was defined as the average GS for all the genes in a module. When the modules of interest were established, the core genes in a module were identified by GS > 0.2 and MS > 0.8. Specifically, in the present study, we also compared the expression levels of the selected core genes between tumor and normal tissues using GEPIA 2.0 (34), which incorporates the transcriptome data of normal pancreas tissue and thus facilitates the identification of differentially expressed genes.

### Trajectory Analysis and Consensus Clustering

The single-cell pseudotime trajectories of the scRNA-seq data were constructed using the Monocle 2 algorithm (35). This algorithm uses a machine learning technique, learning a parsimonious principal graph to reduce the given high-dimensional expression profiles to a low-dimensional space. Single cells were projected to this space and ordered into a trajectory with branch points. For data interpretation, the cells that were located in the same branch were thought to be in the same differentiation state, while cells located in different branches were thought to have different cell differentiation characteristics. Differentially expressed genes between different differentiation states were identified by the R package “limma” (36). Unsupervised consensus clustering based on the differentially expressed genes was conducted using the “ConsensusClusterPlus” package. The clustering procedure included 1,000 iterations, and 80% of the data were sampled in each iteration. The optimal number of clusters was determined by the relative change in the area under the cumulative distribution function (CDF) curves of the consensus score.

### Cell Culture and qRT-PCR

CAFs were first separated and purified from human pancreatic cancer tissues in our laboratory based on the study by Walter et al. and then subjected to immortalization treatment (37). Fresh pancreatic cancer tissue was minced into 1–3 mm^3^ fragments and digested with 0.25% trypsin at 37°C for 30 min. The resulting fragments were centrifuged at 600 × g for 5 min and washed once with Dulbecco's modified Eagle's medium (DMEM) containing 10% fetal bovine serum (FBS). The tissue fragments were then plated and allowed to adhere. After incubation at 37°C for several days, fibroblast outgrowth from the tissue fragments occurred. The fibroblasts were sub-cultured by trypsinization for 2–3 passages until free of epithelial cell contamination and maintained in DMEM supplemented with 10% FBS, 2% penicillin, and streptomycin (Invitrogen). The cells were grown at 37°C in a humidified atmosphere containing 5% CO_2_. CAFs and the pancreatic cancer cell line SW1990 were cultured in DMEM supplemented with 10% FBS. The non-cell supernatant from SW1990 cultures was extracted through centrifugation at 800 rpm/min. Then, CAFs were treated with SW1990-derived supernatant during medium changing. TGF-beta (3 ng/ml) was added to CAF cultures as a positive control.

## Results

### A Meta-Analysis Revealed the Correlation Between Infiltrating Immune Cells and the OS of Patients With PDAC

**Figure 1:**
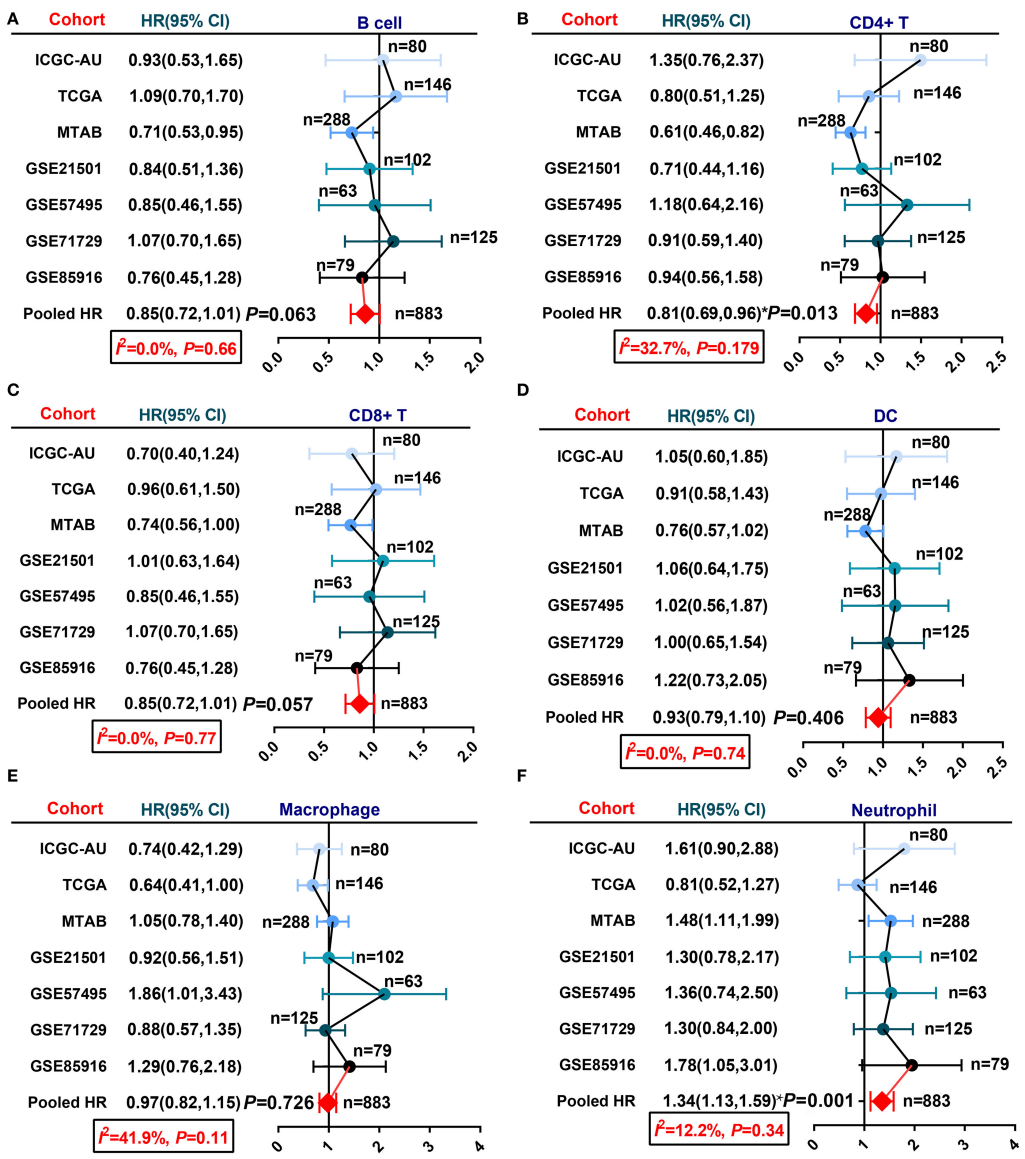
A meta-analysis revealed the association between infiltrated immune cells and PDAC patient OS. **(A)** B cells, **(B)** CD4+ T cells, **(C)** CD8+ T cells, **(D)** dendritic cells, **(E)** macrophages, and **(F)** neutrophils.

In past decades, many approaches have been developed to estimate or quantify the infiltration level of immune cells in tumor tissues, such as immunohistochemical staining and transcriptome-based estimation (16–18, 38, 39). We applied six algorithms, TIMER, CIBERSORT, XCELL, EPIC, QUANTISEQ, and MCP-counter, to estimate the infiltration percentage of immune cells (Supplementary Table 1). Then, we integrated the survival data of patients in seven PDAC cohorts with the infiltration level of each immune cell and performed univariate Cox regression to screen prognosis-related components (Supplementary Table 1). Given the difficulty of pooling the Cox regression-derived HR values due to the large confidence interval (CI) in this case, we performed a meta-analysis using the log-rank test-derived HR values. Six basic immune cell types (CD8+ T cells, CD4+ T cells, B cells, macrophages, neutrophils, and dendritic cells) estimated by the TIMER algorithm were selected for meta-analysis. Null or only minor heterogeneity was detected in the fixed effect model; hence, we generated the results of the meta-analysis with low bias. The results showed that neutrophil infiltration was associated with worse OS for PDAC, while CD8+ T cell, CD4+ T cell, and B cell infiltration was related to prolonged OS for PDAC with marginal statistical significance (Figure 1).

### Single-Cell Sequencing Results Delineated the Heterogeneity of Stromal Cells in the PDAC Microenvironment

**Figure 2:**
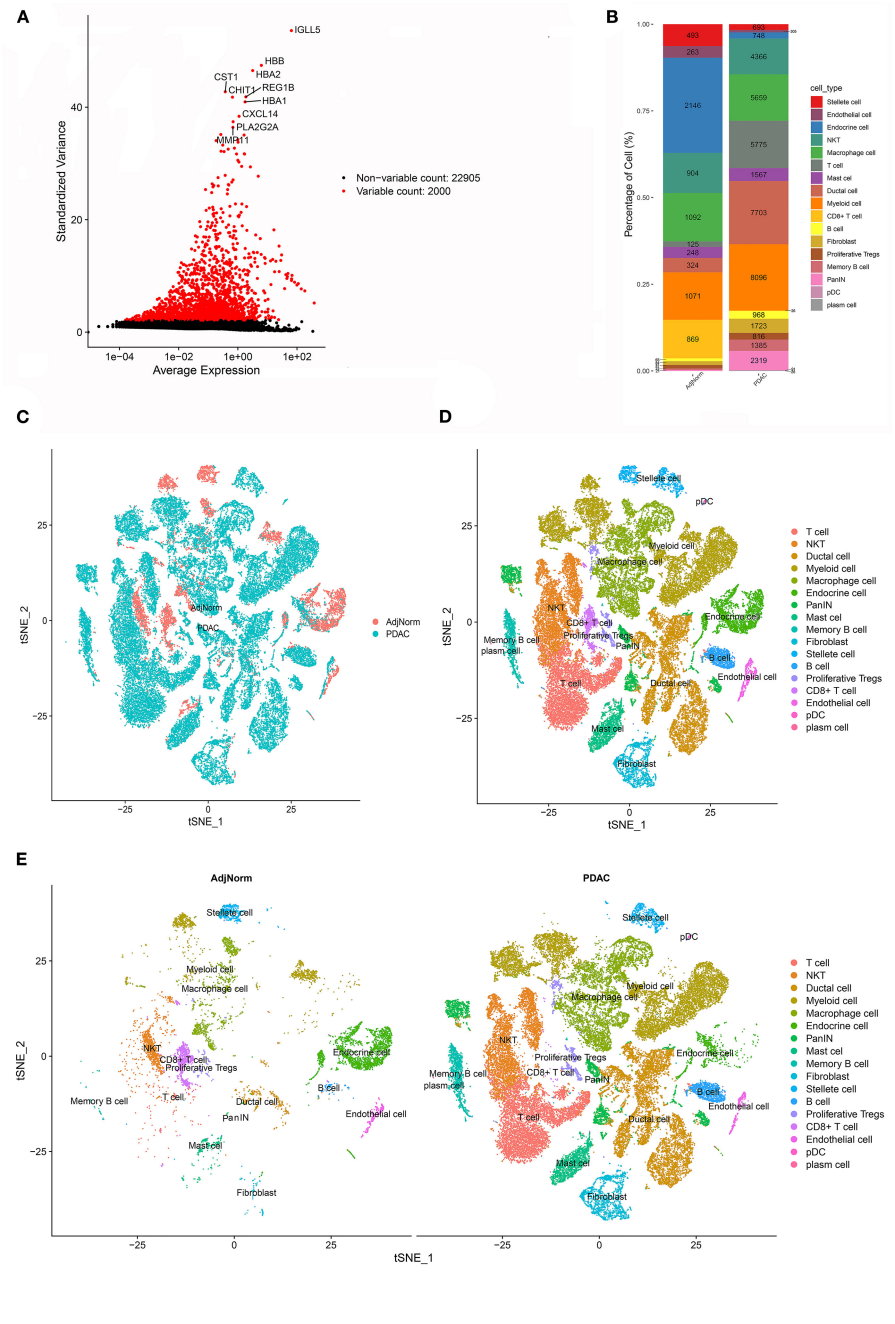
scRNA-seq identified 17 cell types in the PDAC microenvironment. **(A)** The top 2000 variable genes with large, standardized variances were selected for subsequent analysis. **(B)** The percentage of each cell type in PDAC tissues and normal adjacent tissues. **(C–E)** tSNE algorithm classified cell clusters based on transcriptome data.

Interestingly, many theoretical anticancer cells, such as CD8+ T cells, did not show obvious survival relevance in the meta-analysis. We assumed that at least some of these anticancer immune cells were exhausted or experienced differentiation into protumoral cells, which deprived them of their tumor-killing capability. To investigate the alteration of stromal cells in PDAC, we reanalyzed the scRNA-seq dataset and annotated cell types according to their perturbation in the transcriptome. After carrying out the quality control procedures described in the Methods section (Supplementary Figures 1A,B), the top 2,000 variable genes were used in cell clustering (Figure 2A; Supplementary Figures 1C,D), which identified 26 clusters (Supplementary Figures 1E,F) and then classified these clusters into 17 cell types (Supplementary Figure 1G). The proportions of each cell type are shown in Figure 2B, and their clustering distribution is presented in Figures 2C–E; Supplementary Figure 1H. For some clusters, we took a conservative approach and annotated them as basic cell categories, such as T cells and myeloid cells. However, when specific cell markers were highly overexpressed in a cluster, we tended to annotate them into more precise clusters, such as NKT and CD8+ T cells. The significant gene markers are presented in Supplementary Table 2.

### T Cells With a Cytotoxic Signature Predict Prolonged Survival Only in PDAC Patients With the Presence of “Targets” for Pyroptosis and Ferroptosis Induction

**Figure 3:**
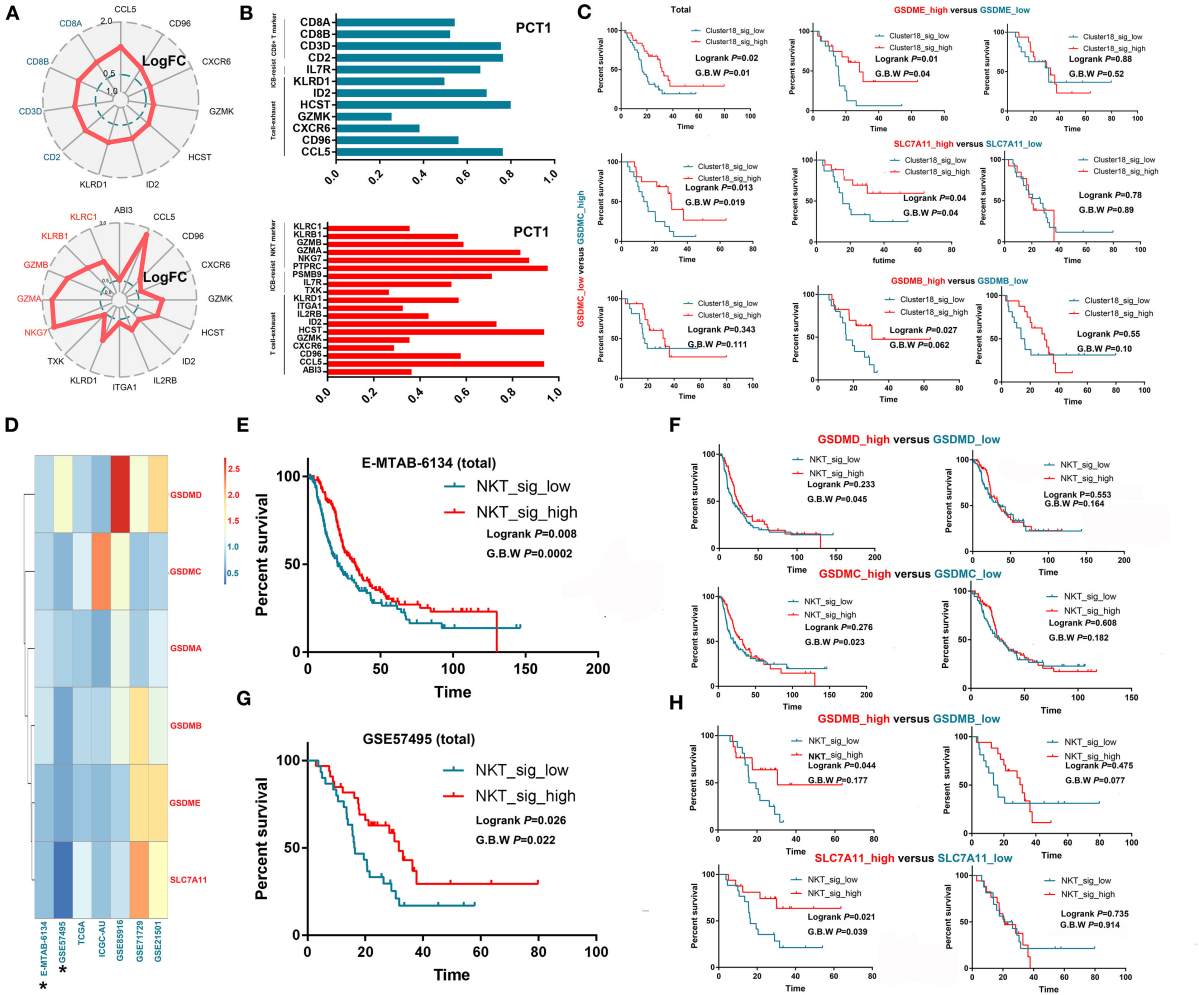
Exhausted cytotoxic T cells predict prolonged survival only in PDACs with the presence of “targets” for pyroptosis and ferroptosis induction. **(A,B)** Intratumorally infiltrated cytotoxic T cells universally expressed exhaustion markers and ICB resistance signatures. LogFC refers to log_2_ (fold change); fold change equals the ratio between the mRNA level of a specific gene in one cell cluster and that in the other cell clusters. PCT1 refers to the percentage of cells that express a specific gene. **(C)** CD8+ T cell infiltration predicts prolonged OS only in samples overexpressing GSDMB, GSDMC, GSDME and SLC7A11, which are targets for cytotoxic T cells to induce pyroptosis and ferroptosis. **(D)** Heatmap showing the distribution of target scores among different PDAC cohorts. **(E–H)** NKT cell infiltration predicts prolonged OS only in samples overexpressing GSDMB-D and SLC7A11, which are targets for cytotoxic T cells to induce pyroptosis and ferroptosis.

NKT and CD8+ T cells are theoretically capable of killing tumor cells through cytotoxic effects; however, most of these cells seem to lose their anticancer ability in the real tumor microenvironment. Through scRNA-seq, we verified that these T cells with a cytotoxic signature universally expressed exhaustion-related or ICB resistance markers (Figures 3A,B), which not only produced immune evasion among tumor cells but also caused a low response to immunotherapy, such as ICB. Then, we tested whether such signatures of exhausted cytotoxic T cells were associated with patient prognoses. As expected, no obvious association was observed between the signature of CD8+ T cells and OS in most cohorts except for GSE57495 (Supplementary Figure 2; Figure 3C). Then, we explored why the signatures of exhausted CD8+ T cells could still predict prolonged OS in some patients. Several recent studies suggested a novel mechanism by which cytotoxic cells trigger tumor cell death, as we reviewed previously (10). These studies demonstrated that cytotoxic T cells, including CD8+ T cells and NKT cells, could kill tumor cells through ferroptosis and pyroptosis induction. Then, we validated this hypothesis in GSE57495, which was the only dataset that showed a correlation between exhausted cytotoxic T cell signatures and patient prognoses. Survival analysis showed that the signature of CD8+ T cells was positively associated with prolonged OS only in samples harboring overexpression of targets for ferroptosis (SLC7A11) and pyroptosis (GSDMB, GSDMC, and GSDME) induction (Figure 3C). We continued to investigate the association between the NKT cell signature and patient OS and found that the NKT signature predicted better OS in only two datasets (E-MTAB-6134 and GSE57495). We generated a new index called the target score, which is defined as $\text{target score}=\frac{\text{HR}(\text{targets high expression})}{\text{HR}(\text{targets low expression})}$. In this equation, HR(targets high expression) reflects the influence of the NKT signature on patients' OS in PDAC samples with overexpression of specific targets for ferroptosis or pyroptosis, while HR (targets low expression) reflects the influence of the NKT signature on patients' OS in PDAC samples with downregulation of specific targets for ferroptosis or pyroptosis. A heatmap was generated to visualize the Target_score of different targets across various datasets (Figure 3D), which suggested universal survival benefits caused by high NKT signature expression in samples with high target expression. Survival analysis further demonstrated that the high expression of some ferroptosis and pyroptosis targets was a precondition for the NKT signature predicting a benefit in terms of OS (Figures 3E–H). Then, we determined the independency of the prognostic implication of the NKT signature from other infiltrated immune cells. Multivariate Cox regression showed that the cytotoxic T cell signature was associated with prolonged OS, independent of other components in the PDAC microenvironment (Supplementary Table 3).

### T Cells With Increased Ribosome-Related Protein Signatures Predict a Better Prognosis in PDAC

**Figure 4:**
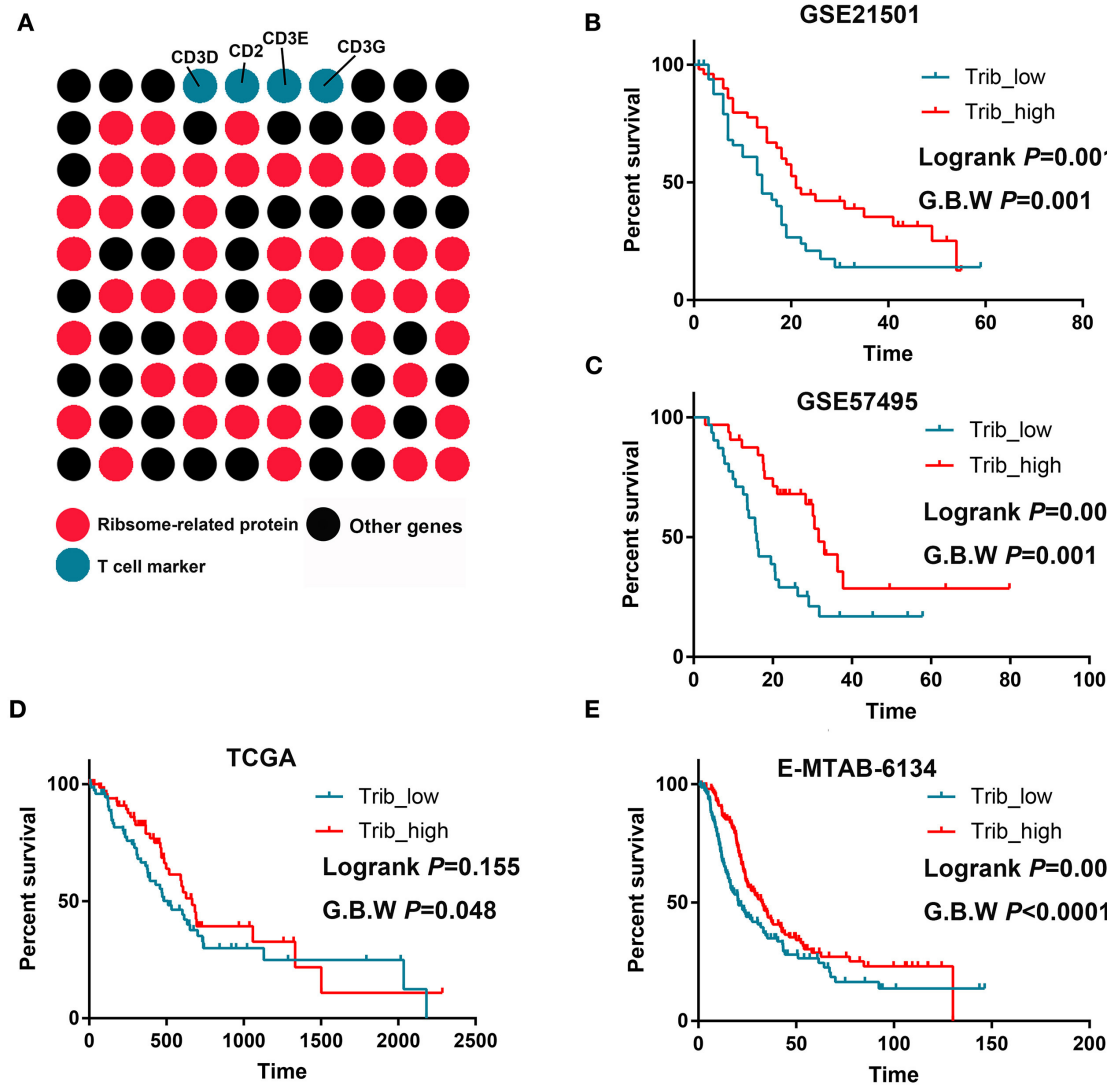
T cells with increased ribosome-related protein signatures predict better prognoses for PDAC. **(A)** Approximately 50% of the markers of this T cell cluster were ribosome-related proteins. **(B–E)** High infiltration of this T cell cluster predicts prolonged OS in GSE21501, GSE57495, TCGA, and MTAB.

An LTB(+)IL-7R(+)CD3(+) cell cluster was identified by scRNA-seq (cluster 0). We analyzed the top 100 upregulated genes in this cluster and showed that 51 genes were ribosome-related proteins (Figure 4A). Then, we performed survival analysis and found that the cluster 0 signatures could predict prolonged OS in PDAC in multiple datasets (Figures 4B–E).

In addition, we investigated the prognostic implications of the three states of B cells in PDAC. Overall, these signatures predict prolonged OS in only some cohorts (Supplementary Figure 3A). Notably, plasma cell infiltration predicted better OS in the three cohorts (MTAB, ICGC, and GSE57495) (Supplementary Figure 3B).

### Increased Hematopoietic Stem Cell (HSC)-Like Signatures Predict Better Prognoses in Patients With PDAC

**Figure 5:**
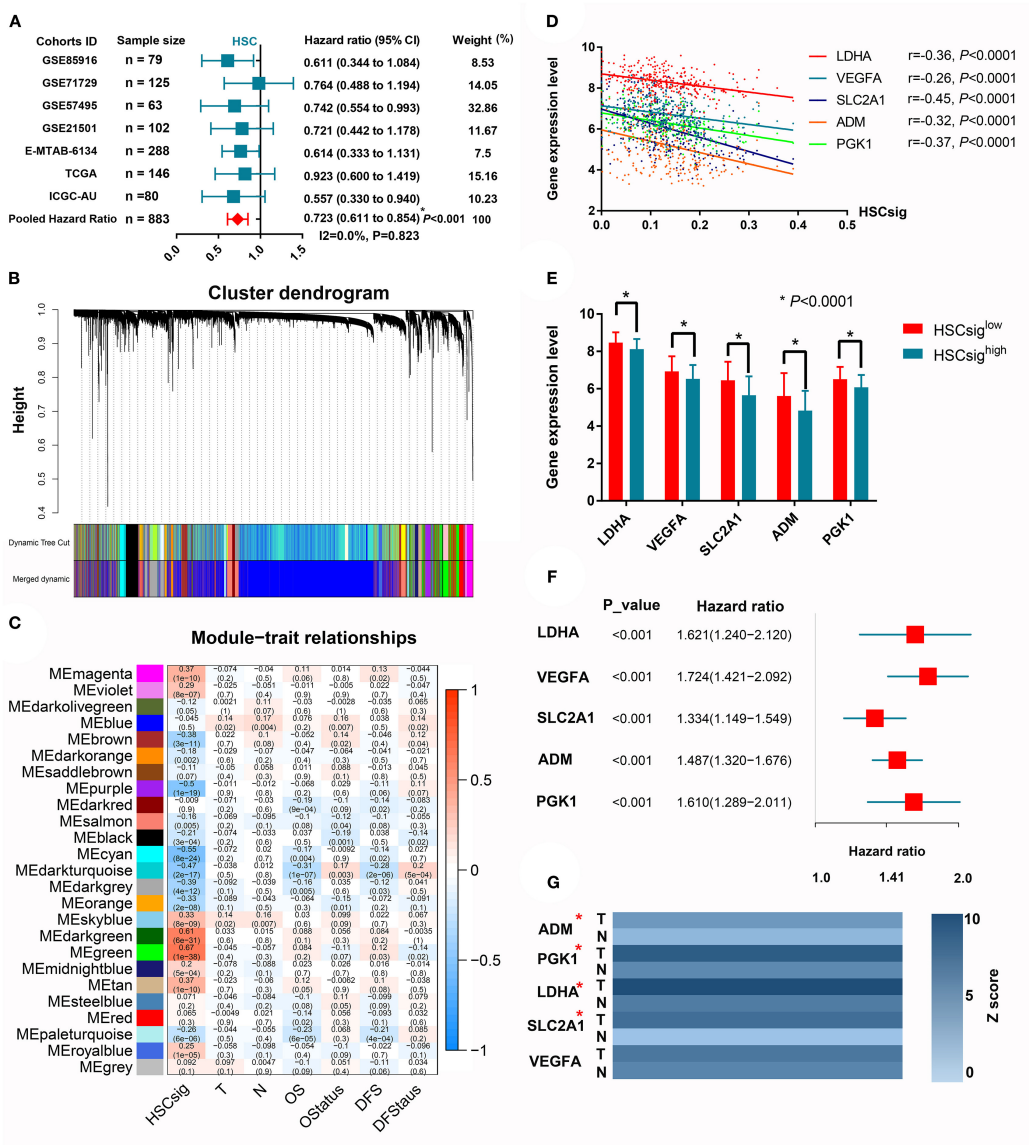
Increased hematopoietic stem cell-like signatures predict better prognoses in patients with PDAC. **(A)** A meta-analysis revealed that HSC-like signatures predict prolonged OS in PDAC patients. **(B)** WGCNA identified gene modules with high coexpression correlation. **(C)** The correlations between 25 coexpressed gene modules and HSC-like signatures and clinical characteristics. **(D)** The linear correlations between the expression of five core genes and HSC-like signatures. **(E)** Comparison of the expression levels between groups with high or low levels of HSC-like signatures. **(F)** The survival implications of five core genes in PDAC. **(G)** The differential expression of core genes between tumor and adjacent normal tissues. ^*^P < 0.05.

The XCELL algorithm calculated the infiltration percentage of HSCs in the PDAC microenvironment; however, no direct evidence indicated the existence of intratumoral HSCs in previous studies, and scRNA-seq was performed here. It is biologically plausible that some cell clusters retain parts of the molecular signatures of HSCs. For example, we found that CD34, as a classical marker of HSCs, was significantly upregulated in cluster 19, which we identified through scRNA-seq (logFC = 1.48). Furthermore, we conducted a meta-analysis to investigate whether the HSC signature was associated with the patient prognosis. The results showed that increased HSC signatures significantly predicted prolonged OS in PDAC (HR = 0.72, 95% CI 0.61–0.85) (Figure 5A). To further analyze the mechanism by which HSC signatures could predict prolonged OS in PDAC, we performed WGCNA to explore the gene modules associated with HSC signatures in the MTAB cohort, which is the largest PDAC cohort with completed transcriptome and follow-up data (Figure 5B). Interestingly, the dark turquoise module was negatively associated with HSC signatures and the OS and DFS of patients (*r* = −0.47, *r* = −0.31, and *r* = −0.28, respectively) (Figure 5C). According to the criteria GS > 0.2 and MS > 0.8, we identified 5 core genes associated with HSC signatures (Figures 5D,E) and patient prognoses (Figure 5F). Given that all five genes were related to unfavorable prognoses, we speculated that these genes may be differentially expressed between tumor and normal tissues. Then, we validated the prognostic implications of these five hub genes in six other cohorts, where each gene was associated with unfavorable OS in at least two validation datasets (Supplementary Figure 4). Using the integrated data of the tumor transcriptome from the TCGA database and the normal pancreas transcriptome from the Genotype-Tissue Expression (GTEx) database, we showed that LDHA, SLC2A1, and PGK1 were upregulated in tumor samples (logFC > 2, *P* < 0.05).

### The Evolutionary Trajectory and Prognostic Implications of Myeloid Cells in PDAC

**Figure 6:**
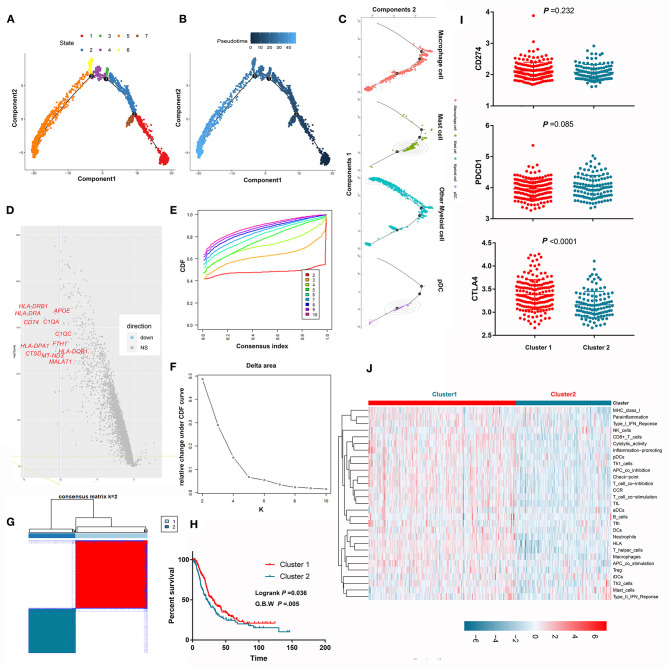
Pseudotime trajectory analysis revealed the evolutionary characteristics of myeloid cells. **(A)** Pseudotime trajectory analysis revealed 7 different states of myeloid cells. **(B)** The gradation of color reflects pseudotime flows. **(C)** The pseudotime trajectory of each cell type in PDAC. **(D)** The volcano plot shows that many antigen-presenting molecules and complement-associated genes were lost along the pseudotime flow. **(E–G)** Unsupervised consensus clustering identified two independent subclusters based on the expression levels of the differentially expressed genes between two cell states spanning the longest pseudotime. **(H)** Survival analysis showed that the prognosis of patients in subcluster 1 was significantly better than that of patients in subcluster 2. **(I)** Comparison the expression of immune-check point between subcluster 1 and subcluster 2. **(J)** Comparison of the activity of 29 immune signatures between subcluster 1 and subcluster 2.

Myeloid cells are important components in the PDAC microenvironment and consist of macrophage cells, myeloid derived suppressive cells, dendritic cells, and granulocytes. Through scRNA-seq, we identified nine myeloid cell clusters (clusters 3–6, 10, 12, 22–23, and 25) and further annotated them as macrophages, mast cells, plasma dendritic cells, and other myeloid cells. Pseudotime trajectory analysis revealed seven different cell states (Figure 6A) and showed the distributions of cell states along with pseudotime flows (Figure 6B). We also mapped cell classifications to pseudotime trajectories (Figure 6C). The difference in the transcriptome between the two cell states with the longest pseudotime span was compared, wherein many antigen-presenting molecules and complement-associated genes were lost along the pseudotime flow (Figure 6D). Unsupervised consensus clustering identified two independent clusters based on the pseudotime-related differentially expressed genes in the MTAB cohort (Figures 6E–G). Notably, the OS of patients in cluster 1 was significantly better than that of patients in cluster 2 (*P* < 0.05) (Figure 6H). This result was marginally confirmed in the ICGC cohort (Supplementary Figure 5). Next, we compared the activity of 29 immune signatures between cluster 1 and cluster 2. The results showed that cluster 1 harbored more activated immune signatures, which may account for its survival advantage (Figures 6I,J; Supplementary Figure 4).

### Differentially Expressed Genes Between CAFs in PDAC Tissues and Fibroblasts in Normal Pancreas Tissues

**Figure 7:**
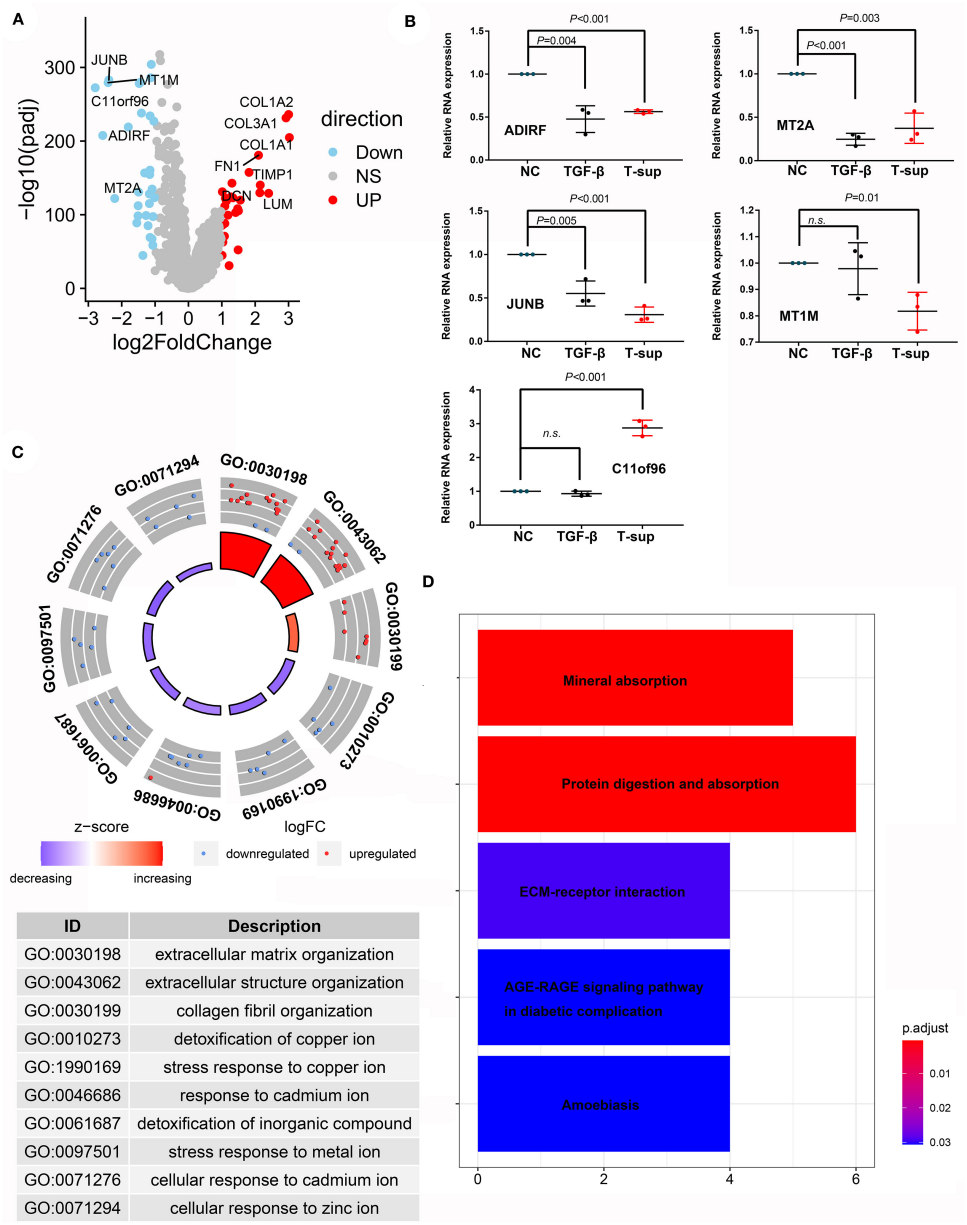
Differences in the transcriptome between CAFs and fibroblasts/PSCs. **(A)** Volcano plot showing differentially expressed genes between CAFs and fibroblasts/PSCs in PDAC tissues and adjacent normal tissues. **(B)** Validation of the downregulated genes in CAFs using tumor supernatant or TGF-beta followed by qPCR. **(C,D)** GO and KEGG analyses revealed the functions of the differentially expressed genes.

Fibroblasts or pancreatic satellite cells exist in normal pancreatic tissues; however, many studies have suggested that the formation of tumors alters the states and function of these cells. Through scRNA-seq analysis, we compared the mRNA expression landscape of fibroblasts and satellite cells between PDAC and normal pancreas tissues (Figure 7A). We found that multiple genes involved in stromal formation, such as COL1A2, COL3A1, COL1A1, FN1, TIMP1, DCN, and LUM, were upregulated in CAFs (logFC > 2, adjusted *P* < 0.05). However, the genes that were downregulated in CAFs have rarely been investigated in CAFs. Hence, we investigated whether PDAC cells could induce alterations in these genes using *in vitro* experiments. Given that TGF-beta is an important mediator regulating the crosstalk between CAFs and PDAC cells, we also established a positive control group using TGF-beta to mimic the activated states of CAFs. Our results showed that the relative mRNA expression of ADIRF, MT2A, MT1M, and JUNB was downregulated after TGF-beta treatment and/or tumor stimulation, while the expression level of C11orf96 was upregulated, even after tumor stimulation (Figure 7B). Gene Ontology (GO) analysis indicated that the upregulated genes in CAFs were mainly related to the extracellular matrix and structural organization, while the downregulated genes in CAFs were associated with the response to mental ions (Figure 7C). Kyoto Encyclopedia of Genes and Genomes (KEGG) analysis also showed that the differentially expressed genes were enriched in mineral absorption and the extracellular-receptor interaction (Figure 7D).

## Discussion

The treatment modalities for multiple cancers, except for PDAC, have entered a new era of targeted and immunological therapy (40). A stringent obstacle in optimizing the efficacy of PDAC treatment is its immunosuppressive and desmoplastic microenvironment, which causes difficulties in drug delivery and low responses to targeted therapy, including ICI-based immunotherapy (41). The key to exploring optimized treatment modalities is the comprehension of the intratumoral heterogeneity of PDAC. Next-generation sequencing (NGS) techniques have led to a rush of researchers exploring cancer genomics or transcriptomes (42). Although NGS interprets numerous biological behaviors of PDAC by defining the dysregulation of oncogenic or tumor-suppressive pathways, it is hard for NGS to decipher the role of each cell type in PDAC development, given that bulk sequencing only reflects the average levels of the tissue being detected (43). Even the same cell type sometimes has a different state and manifests distinguished functions. The development of scRNA-seq has provided researchers with an opportunity to explore intratumoral heterogeneity (44). The expression levels of markers could reflect the infiltration of specific cell types in tumor tissues using algorithms such as ssGSEA and TIMER. In addition, the correlation between the relative infiltration level of a specific cell type and patient survival could be established with complete follow-up data in multiple PDAC cohorts.

In the present study, we first observed the paradox that multiple theoretically tumor-suppressive cell types were not associated with patient prognoses. Then, we tried to explore the underlying mechanism by analyzing the intratumoral heterogeneity in PDAC using scRNA-seq data and 7 PDAC cohorts with bulk sequencing. We found that cytotoxic T cells, including CD8+ T cells and NKT cells, predict prolonged OS only in samples with overexpression of targets for pyroptosis and ferroptosis induction, which was the recently reported potential mechanism by which cytotoxic T cells mediate tumor cell killing (8, 11, 45–47). In addition, a specific state of T cells with overexpression of ribosome-related proteins is associated with a better prognosis. Previous studies have shown the importance of function-intact ribosomes in allowing T cells to execute immune effects (48, 49), while ribosome-targeting antibiotics impair T cell effector function and ameliorate autoimmunity by blocking mitochondrial protein synthesis (50). Hence, maintaining normal ribosome function in intratumoral T cells may contribute to their antitumor efficacy and further improve patient prognoses. In addition, an HSC-like signature predicts better OS in PDAC. WGCNA identified 5 hub genes (LDHA, VEGFA, SLC2A1, ADM, and PGK1) whose downregulation may mediate the observed survival benefits of the HSC-like signature. Interestingly, among these core genes, SLC2A1, LDHA, and PGK1 are classical oncogenic glycolytic enzymes in PDAC (51–53), and glycolytic products such as lactate acids could upregulate the level of VEGFA (54), suggesting that the negative correlation between the HSC-like signature and glycolytic activity may account for the survival benefit associated with high HSC-like signature expression. Moreover, pseudotime trajectory analysis uncovered myeloid cells evolutionarily consisting of 7 states, and antigen-presenting molecules and complement-associated genes were lost with the pseudotime flow. Consensus clustering based on the differentially expressed genes between two states harboring the longest pseudotime span identified two PDAC groups with prognostic differences, and more infiltrated immune cells and activated immune signatures may account for the survival benefits.

This study has some strengths that are noteworthy. On one hand, we integrated the scRNA-seq and bulk sequencing data from 7 PDAC cohorts with complete follow-up data to investigate the prognostic implications of cell components, which is a heavy task with a large sample size. Using the cell markers identified in single-cell sequencing for the same cancer type to calculate the cell fractions in tissues through bulk sequencing has distinguished benefits in avoiding the bias derived from cell heterogeneity among different cancer types. In addition, we yielded several findings that might be implicated for future study and clinical translation. For example, we proposed that cytotoxic T cells, including CD8+ T cells and NKT cells, predict prolonged OS only in samples with overexpression of targets for pyroptosis and ferroptosis induction. Given that we have shown that most intratumorally infiltrated T cells were exhausted using scRNA-seq, this novel tumor-killing approach might also help theoretically exhausted T cells defend against tumor cells. Certainly, the present manuscript also has several limitations. On one hand, we analyzed only transcriptome data; however, proteome data would be an appropriate supplement for validating our conclusions. On the other hand, this is a horizontal bioinformatics study lacking longitudinal mechanistic investigation. Notably, certain signatures predict prolonged OS only in some cohorts not in all cohorts. Several reasons could explain it as follows. First, the transcriptome of tissue samples is easily disturbed, especially when preservation measures are inappropriate. Additionally, the inherent systemic error in RNA sequencing also leads to some unexpected bias. Therefore, the expression levels of some genes may not be accurate in all cohorts. Second, the follow-up OS was in fact influenced by many factors, such as chemotherapy modalities, surgical factors, loss of follow-up bias, etc. As a result, even if a gene is associated with the pathophysiological behavior of PDAC, the association of its gene expression level may not tightly reflect OS in some cases. Third, we defined signature_high and signature_low groups based on the median value, which is a common method that has been widely applied in published studies (55–58).

In conclusion, this study systematically investigated the prognostic implications of the components of the PDAC tumor microenvironment by integrating single-cell sequencing and bulk sequencing, and future studies are expected to develop novel targeted agents for PDAC treatment.

## Data Availability Statement

The original contributions presented in the study are included in the article/Supplementary Material, further inquiries can be directed to the corresponding authors.

## Ethics Statement

This study was approved by the Institutional Research Ethics Committee of Fudan University Shanghai Cancer Center (FUSCC), and written informed consent was obtained from all patients prior to the investigation.

## Author Contributions

RT and XL performed the bioinformatic analysis. WW and JH were in charge of the statistical analysis. JX, CL, and JL checked the tables and figures. BZ, XY, and SS designed the study.

## Conflict of Interest

The authors declare that the research was conducted in the absence of any commercial or financial relationships that could be construed as a potential conflict of interest.

## Supplementary Material

Supplementary Figure 1 scRNA-seq identified 26 independent cell clusters in PDAC. **(A)** The mitochondrial RNA level is not associated with increased read counts, while qualified RNA is positively correlated with increased read counts. **(B)** The distribution of qualified RNA, total read counts and mitochondrial RNA levels in tumor and adjacent tissues. **(C)** The standard deviation and the number of principal components. **(D)** PDAC and adjacent normal tissues could be distinguished by two principal components. **(E,F)** The tSNE and UMAP algorithms classified PDAC samples into 26 independent clusters. **(G)** The 26 clusters were annotated into 17 cell types according to specific cell markers.

Supplementary Figure 2 Null survival benefits of CD8+ T cells were detected in six PDAC cohorts.

Supplementary Figure 3 The association between B cell signatures and the OS of PDAC patients. **(A)** The whole landscape. **(B)** The survival curve showed results with statistical significance.

Supplementary Figure 4 Validation of the prognostic implications of the hub genes identified from WGCNA.

Supplementary Figure 5 Validation of the consensus clustering results in another dataset (ICGC). **(A–C)** Unsupervised consensus clustering identified two independent subclusters based on the expression levels of the differentially expressed genes between two cell states spanning the longest pseudotime. **(D)** Survival analysis showed that the prognosis of patients in subcluster 1 was marginally better than that of patients in subcluster 2.

Supplementary Table 1 The raw data and univariate COX analysis for the 119 cell signatures and PDACs' survival in 7 cohorts.

Supplementary Table 2 The gene markers of different cell clusters.

Supplementary Table 3 Multivariate cox regression validates the association between OS and cytotoxic T cell signatures in targets_high group is independent of other infiltrated cell components.

## References

[1] Siegel RL Miller KD Jemal A. Cancer statistics, 2020. CA Cancer J Clin. (2020) 70:7–30. 10.3322/caac.21590 31912902

[2] Moore A Donahue T. Pancreatic cancer. JAMA. (2019) 322:1426. 10.1001/jama.2019.14699 31593274 PMC7021307

[3] Shi S Hua J Liang C Meng Q Liang D Xu J. Proposed modification of the 8th edition of the AJCC staging system for pancreatic ductal adenocarcinoma. Ann Surg. (2019) 269:944–50. 10.1097/SLA.0000000000002668 29334560

[4] Emens LA Ascierto PA Darcy PK Demaria S Eggermont AMM Redmond WL. Cancer immunotherapy: opportunities and challenges in the rapidly evolving clinical landscape. Eur J Cancer. (2017) 81:116–29. 10.1016/j.ejca.2017.01.035 28623775

[5] Segal NH Diaz LA Jr Moskovitz J Moy J Ferris RL. Immunotherapy for head and neck squamous cell carcinoma. Nat Rev Gastroenterol Hepatol. (2018) 20:22. 10.1007/s11912-018-0654-5 PMC5835060 29502288

[6] Ganesh K Stadler ZK Cercek A Mendelsohn RB Shia J. Immunotherapy in colorectal cancer: rationale, challenges and potential. Nat Rev Gastroenterol Hepatol. (2019) 16:361–75. 10.1038/s41575-019-0126-x 30886395 PMC7295073

[7] Balachandran VP Beatty GL Dougan SK. Broadening the impact of immunotherapy to pancreatic cancer: challenges and opportunities. Gastroenterology. (2019) 156:2056–72. 10.1053/j.gastro.2018.12.038 30660727 PMC6486864

[8] Wang W Green M Choi JE Gijón M Kennedy PD Johnson JK. CD8(+) T cells regulate tumour ferroptosis during cancer immunotherapy. Nature. (2019) 569:270–4. 10.1038/s41586-019-1170-y 31043744 PMC6533917

[9] Xi G Gao J Wan B Zhan P Xu W Lv T. GSDMD is required for effector CD8(+) T cell responses to lung cancer cells. Int Immunopharmacol. (2019) 74:105713. 10.1016/j.intimp.2019.105713 31276977

[10] Tang R Xu J Zhang B Liu J Liang C Hua J. (2020). Ferroptosis, necroptosis, and pyroptosis in anticancer immunity. J Hematol Oncol. (2020) 13:110. 10.1186/s13045-020-00946-7 32778143 PMC7418434

[11] Zhang Z Zhang Y Xia S Kong Q Li S Liu X. Gasdermin E suppresses tumour growth by activating anti-tumour immunity. Nature. (2020) 579:415–20. 10.1038/s41586-020-2071-9 32188940 PMC7123794

[12] Qin X Yan M Wang X Xu Q Wang X Zhu X. Cancer-associated fibroblast-derived IL-6 promotes head and neck cancer progression via the osteopontin-NF-kappa b signaling pathway. Theranostics. (2018) 8:921–40. 10.7150/thno.22182 29463991 PMC5817102

[13] Wen S Hou Y Fu L Xi L Yang D Zhao M. Cancer-associated fibroblast. (CAF)-derived IL32 promotes breast cancer cell invasion and metastasis via integrin β3-p38 MAPK signalling. Cancer Lett. (2019) 442:320–32. 10.1016/j.canlet.2018.10.015 30391782

[14] Ligorio M Sil S Malagon-Lopez J Nieman LT Misale S Di Pilato M. Stromal microenvironment shapes the intratumoral architecture of pancreatic cancer. Nat Biotechnol. (2019) 178:e127. 10.1016/j.cell.2019.05.012 31155233 PMC6697165

[15] Moncada R Barkley D. Integrating microarray-based spatial transcriptomics and single-cell RNA-seq reveals tissue architecture in pancreatic ductal adenocarcinomas. Nat Biotechnol. (2020). 38:333–42. 10.1038/s41587-019-0392-8 31932730

[16] Aran D Hu Z Butte AJ. xCell: digitally portraying the tissue cellular heterogeneity landscape. Genome Biol. (2017) 18:220. 10.1186/s13059-017-1349-1 29141660 PMC5688663

[17] Li T Fan J Wang B Traugh N Chen Q Liu JS. TIMER: a web server for comprehensive analysis of tumor-infiltrating immune cells. Cancer Res. (2017) 77:e108–10. 10.1158/0008-5472.CAN-17-0307 29092952 PMC6042652

[18] Chen B Khodadoust MS Liu CL Newman AM Alizadeh AA. Profiling tumor infiltrating immune cells with CIBERSORT. Methods Mol Biol. (2018) 1711:243–59. 10.1007/978-1-4939-7493-1_12 29344893 PMC5895181

[19] Philip M Schietinger A. Heterogeneity and fate choice: T cell exhaustion in cancer and chronic infections. Curr Opin Immunol. (2019) 58:98–103. 10.1016/j.coi.2019.04.014 31181510 PMC7608527

[20] van der Leun AM Thommen DS. CD8(+) T cell states in human cancer: insights from single-cell analysis. Nat Rev Cancer. (2020) 20:218–32. 10.1038/s41568-019-0235-4 32024970 PMC7115982

[21] Yuan A Hsiao YJ Chen HY Chen HW Ho CC Chen YY. Opposite effects of M1 and M2 macrophage subtypes on lung cancer progression. Sci Rep. (2015) 5:14273. 10.1038/srep14273 26399191 PMC4585843

[22] Orecchioni M Ghosheh Y Pramod AB Ley K. Macrophage polarization: different gene signatures in M1(LPS+) vs. classically and M2(LPS-) vs. alternatively activated macrophages. Front Immunol. (2019) 10:1084. 10.3389/fimmu.2019.01084 31178859 PMC6543837

[23] Li T Fu J Zeng Z Cohen D Li J Chen Q. TIMER2.0 for analysis of tumor-infiltrating immune cells. Nucleic Acids Res. (2020) 48:W509–14. 10.1093/nar/gkaa407 32442275 PMC7319575

[24] Stuart T Butler A Hoffman P Hafemeister C Papalexi E Mauck WM. Comprehensive integration of single-cell data. Cell. (2019) 177:e1821. 10.1016/j.cell.2019.05.031 PMC6687398 31178118

[25] Lall S Sinha D Bandyopadhyay S Sengupta D. Structure-aware principal component analysis for single-cell RNA-seq data. J Comput Biol. (2018) 25:1365–73. 10.1089/cmb.2018.0027 30133312

[26] Satija R Farrell JA Gennert D Schier AF Regev A. Spatial reconstruction of single-cell gene expression data. Nat Biotechnol. (2015) 33:495–502. 10.1038/nbt.3192 25867923 PMC4430369

[27] Aran D Looney AP Liu L Wu E Fong V Hsu A. Reference-based analysis of lung single-cell sequencing reveals a transitional profibrotic macrophage. Nat Immunol. (2019) 20:163–72. 10.1038/s41590-018-0276-y 30643263 PMC6340744

[28] Zhang X Lan Y Xu J Quan F Zhao E Deng C. CellMarker: a manually curated resource of cell markers in human and mouse. Nucleic Acids Res. (2019) 47:D721–8. 10.1093/nar/gky900 30289549 PMC6323899

[29] Peng J Sun BF Chen CY Zhou JY Chen YS. Single-cell RNA-seq highlights intra-tumoral heterogeneity and malignant progression in pancreatic ductal adenocarcinoma. Cell Res. (2019) 29:725–38. 10.1038/s41422-019-0195-y 31273297 PMC6796938

[30] Wu W Elyada E Bolisetty M Laise P Flynn WF. Cross-species single-cell analysis of pancreatic ductal adenocarcinoma reveals antigen-presenting cancer-associated fibroblasts. Cell Res. (2019) 9:1102–23. 10.1158/2159-8290.CD-19-0094 31197017 PMC6727976

[31] Schlesinger Y Yosefov-Levi O Kolodkin-Gal D Granit RZ Peters L Kalifa R. Single-cell transcriptomes of pancreatic preinvasive lesions and cancer reveal acinar metaplastic cells' heterogeneity. Nat Biotechnol. (2020) 11:4516. 10.1038/s41467-020-18207-z 32908137 PMC7481797

[32] Hänzelmann S Castelo R Guinney J. GSVA: gene set variation analysis for microarray and RNA-seq data. BMC Bioinformat. (2013) 14:7. 10.1186/1471-2105-14-7 23323831 PMC3618321

[33] Langfelder P Horvath S. WGCNA: an R package for weighted correlation network analysis. BMC Bioinformat. (2008) 9:559. 10.1186/1471-2105-9-559 19114008 PMC2631488

[34] Tang Z Kang B Li C Chen T Zhang Z. GEPIA2: an enhanced web server for large-scale expression profiling and interactive analysis. Nucleic Acids Res. (2019) 47:W556–60. 10.1093/nar/gkz430 31114875 PMC6602440

[35] Qiu X Mao Q Tang Y Wang L Chawla R Pliner HA. Reversed graph embedding resolves complex single-cell trajectories. Nat Methods. (2017). 14:979–82. 10.1038/nmeth.4402 28825705 PMC5764547

[36] Ritchie ME Phipson B Wu D Hu Y Law CW Shi W. limma powers differential expression analyses for RNA-sequencing and microarray studies. Nucleic Acids Res. (2015) 43:e47. 10.1093/nar/gkv007 25605792 PMC4402510

[37] Walter K Omura N Hong SM Griffith M Goggins M. Pancreatic cancer associated fibroblasts display normal allelotypes. Cancer Biol Ther. (2008) 7:882–8. 10.4161/cbt.7.6.5869 18344687 PMC2692624

[38] Scanziani E. Immunohistochemical staining of fixed tissues. Methods Mol Biol. (1998) 104:133–40. 10.1385/0-89603-525-5:133 9711649

[39] Otali D Fredenburgh J Oelschlager DK Grizzle WE. A standard tissue as a control for histochemical and immunohistochemical staining. Biotech Histochem. (2016) 91:309–26. 10.1080/10520295.2016.1179342 27149658 PMC5338041

[40] Gotwals P Cameron S Cipolletta D Cremasco V Crystal A Hewes B. Prospects for combining targeted and conventional cancer therapy with immunotherapy. Nat Rev Cancer. (2017) 17:286–301. 10.1038/nrc.2017.17 28338065

[41] Li KY Yuan JL Trafton D Wang JX Niu N Yuan CH. Pancreatic ductal adenocarcinoma immune microenvironment and immunotherapy prospects. Chronic Dis Transl Med. (2020) 6:6–17. 10.1016/j.cdtm.2020.01.002 32226930 PMC7096327

[42] Kamps R Brandão RD Bosch BJ Paulussen AD Xanthoulea S Blok MJ. Next-generation sequencing in oncology: genetic diagnosis, risk prediction and cancer classification. Int J Mol Sci. (2017) 18:308. 10.3390/ijms18020308 28146134 PMC5343844

[43] Morganti S Tarantino P Ferraro E D'Amico P Duso BA Curigliano G. Next generation sequencing (NGS): a revolutionary technology in pharmacogenomics and personalized medicine in cancer. Adv Exp Med Biol. (2019) 1168:9–30. 10.1007/978-3-030-24100-1_2 31713162

[44] Papalexi E Satija R. Single-cell RNA sequencing to explore immune cell heterogeneity. Nat Rev Immunol. (2018) 18:35–45. 10.1038/nri.2017.76 28787399

[45] Minton K. Pyroptosis heats tumour immunity. Nat Rev Immunol. (2020) 20:274–5. 10.1038/s41577-020-0297-2 32203327

[46] Nicolai CJ Raulet DH. Killer cells add fire to fuel immunotherapy. Science. (2020) 368:943–4. 10.1126/science.abc2502 32467380

[47] Zhou Z He H. Granzyme A from cytotoxic lymphocytes cleaves GSDMB to trigger pyroptosis in target cells. Science. (2020) 368:eaaz7548. 10.1126/science.aaz7548 32299851

[48] Tan TCJ Knight J Sbarrato T Dudek K Willis AE Zamoyska R. Suboptimal T-cell receptor signaling compromises protein translation, ribosome biogenesis, and proliferation of mouse CD8 T cells. Proc Natl Acad Sci USA. (2017) 114:E6117–26. 10.1073/pnas.1700939114 28696283 PMC5544288

[49] Wolf T Jin W Zoppi G Vogel IA Akhmedov M Bleck CKE. Dynamics in protein translation sustaining T cell preparedness. Nat Immunol. (2020) 21:927–37. 10.1038/s41590-020-0714-5 32632289 PMC7610365

[50] Almeida L Dhillon-LaBrooy A Castro CN Adossa N Carriche GM Guderian M. Ribosome-targeting antibiotics impair T cell effector function and ameliorate autoimmunity by blocking mitochondrial protein synthesis. Immunity. (2020) 92:1408–19. 10.1101/832956 33238133 PMC7837214

[51] Nagarajan A Dogra SK Sun L Gandotra N Ho T Cai G. Paraoxonase 2 facilitates pancreatic cancer growth and metastasis by stimulating GLUT1-mediated glucose transport. Mol Cell. (2017) 67:e686. 10.1016/j.molcel.2017.07.014 28803777 PMC5567863

[52] Fleming JB Fukuhisa H Seki N Idichi T Kurahara H Yamada Y. Gene regulation by antitumor miR-130b-5p in pancreatic ductal adenocarcinoma: the clinical significance of oncogenic EPS8. J Hum Genet. (2019) 64:521–34. 10.1038/s10038-019-0584-6 30858505

[53] Liang C Shi S Qin Y Meng Q Hua J Hu Q. Localisation of PGK1 determines metabolic phenotype to balance metastasis and proliferation in patients with SMAD4-negative pancreatic cancer. Gut. (2020) 69:888–900. 10.1136/gutjnl-2018-317163 31611300

[54] Song J Lee K Park SW Chung H Jung D Na YR. Lactic acid upregulates VEGF expression in macrophages and facilitates choroidal neovascularization. Invest Ophthalmol Vis Sci. (2018) 59:3747–54. 10.1167/iovs.18-23892 30046816

[55] Brooks JM Menezes AN Ibrahim M Archer L Lal N Bagnall CJ. Development and validation of a combined hypoxia and immune prognostic classifier for head and neck cancer. Clin Cancer Res. (2019) 25:5315–28. 10.1158/1078-0432.CCR-18-3314 31182433

[56] Karasinska JM Topham JT Kalloger SE Jang GH Denroche RE Culibrk L. Altered gene expression along the glycolysis-cholesterol synthesis axis is associated with outcome in pancreatic cancer. Clin Cancer Res. (2020) 26:135–46. 10.1158/1078-0432.CCR-19-1543 31481506

[57] Lv J Zhu Y Ji A Zhang Q Liao G. Mining TCGA database for tumor mutation burden and their clinical significance in bladder cancer. Biosci Rep. (2020) 40:BSR20194337. 10.1042/BSR20194337 32239176 PMC7178217

[58] Zhang M Wang X Chen X Guo F Hong J. Prognostic value of a stemness index-associated signature in primary lower-grade glioma. Front Genet. (2020) 11:441. 10.3389/fgene.2020.00441 32431729 PMC7216823

