# Supplementary figures and images for: Deciphering the Prognostic Implications of the Components and Signatures in the Immune Microenvironment of Pancreatic Ductal Adenocarcinoma
Source: Front Immunol. 2021 Mar 10;12:648917. doi: 10.3389/fimmu.2021.648917 (PMC7987951; doi:10.3389/fimmu.2021.648917)

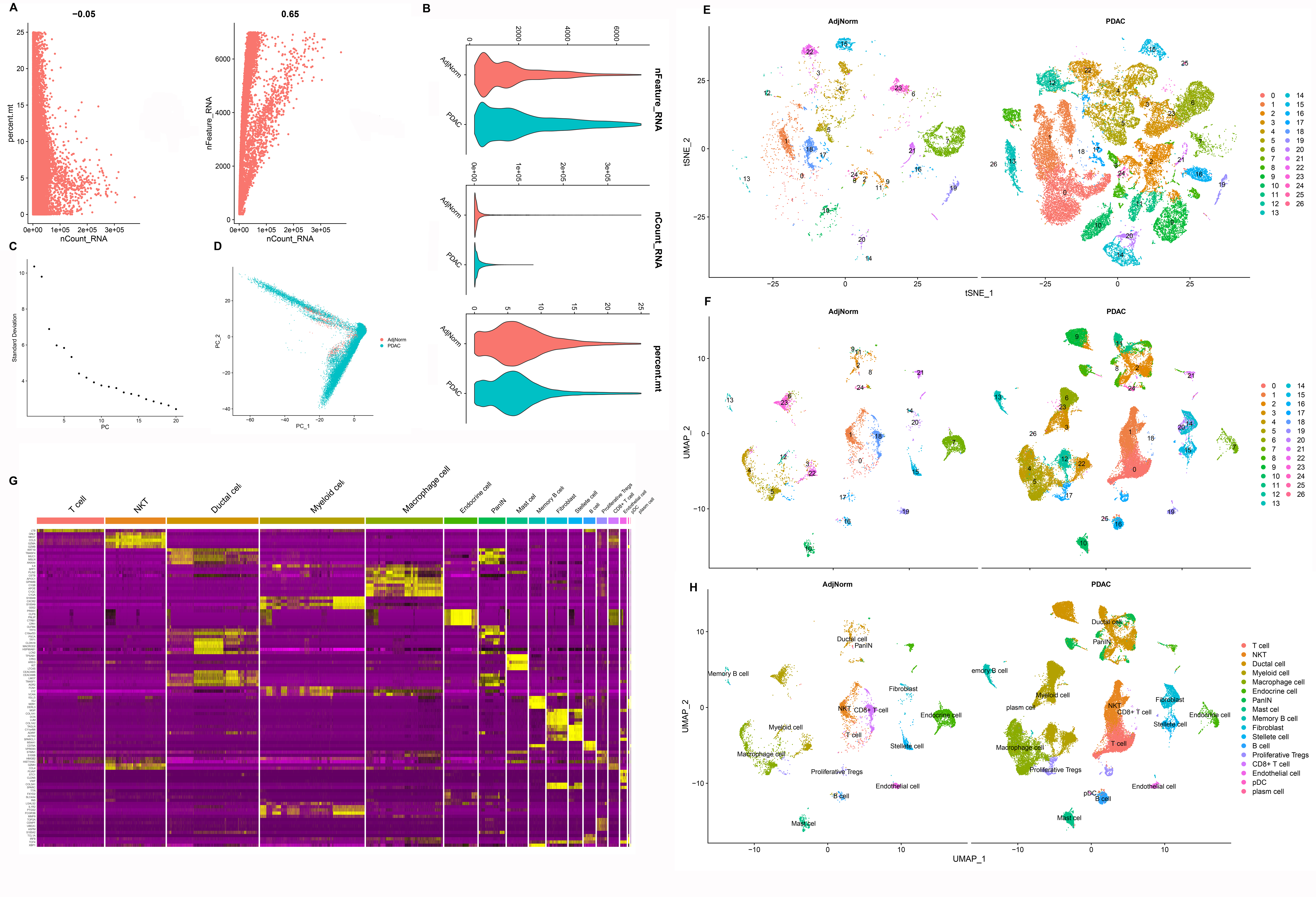

Supplement: Supplementary Figure 1 — scRNA-seq identified 26 independent cell clusters in PDAC. (A) The mitochondrial RNA level is not associated with increased read counts, while qualified RNA is positively correlated with increased read counts. (B) The distribution of qualified RNA, total read counts and mitochondrial RNA levels in tumor and adjacent tissues. (C) The standard deviation and the number of principal components. (D) PDAC and adjacent normal tissues could be distinguished by two principal components. (E,F) The tSNE and UMAP algorithms classified PDAC samples into 26 independent clusters. (G) The 26 clusters were annotated into 17 cell types according to specific cell markers. [file Image_1.JPEG]

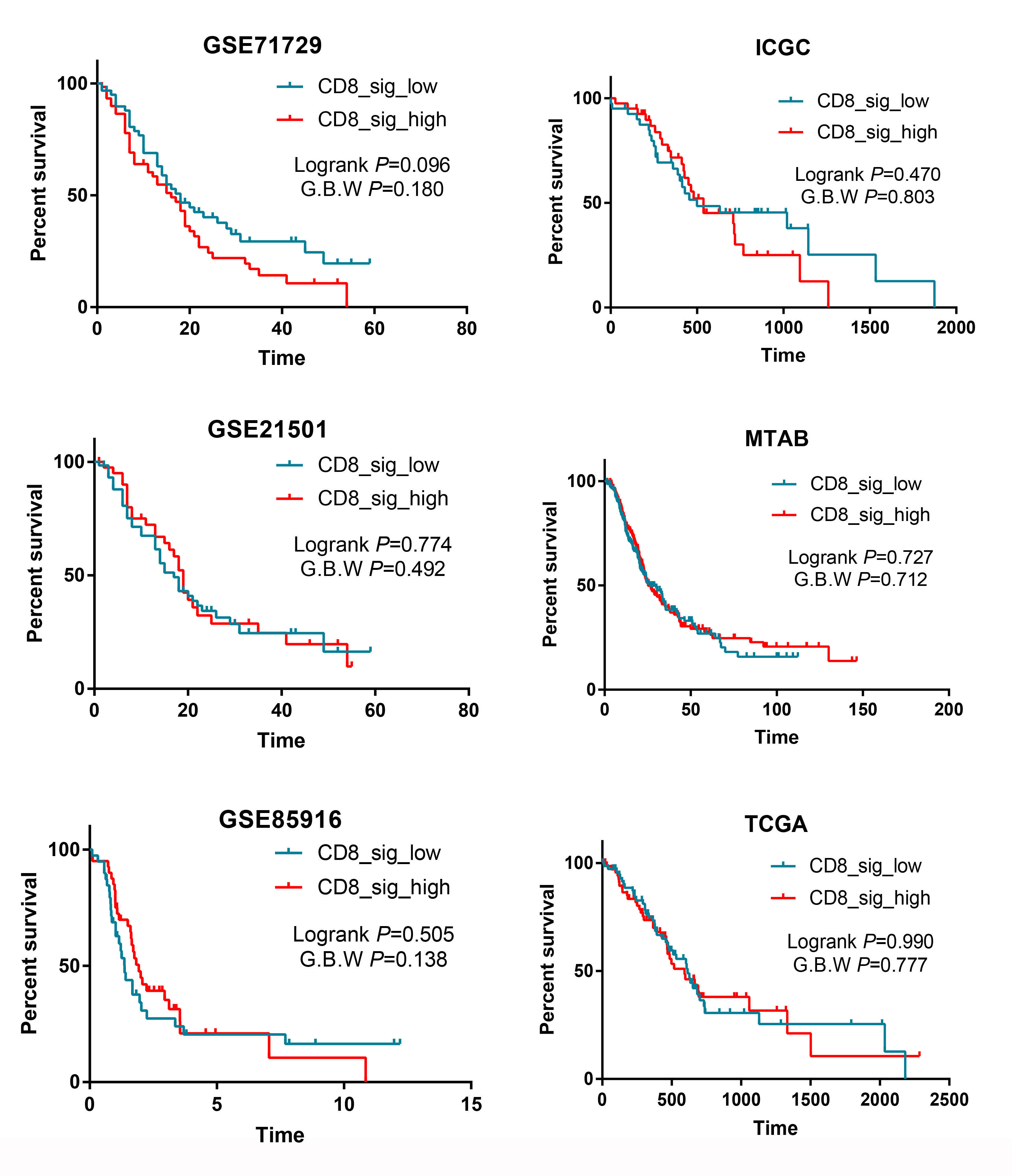

Supplement: Supplementary Figure 2 — Null survival benefits of CD8+ T cells were detected in six PDAC cohorts. [file Image_2.JPEG]

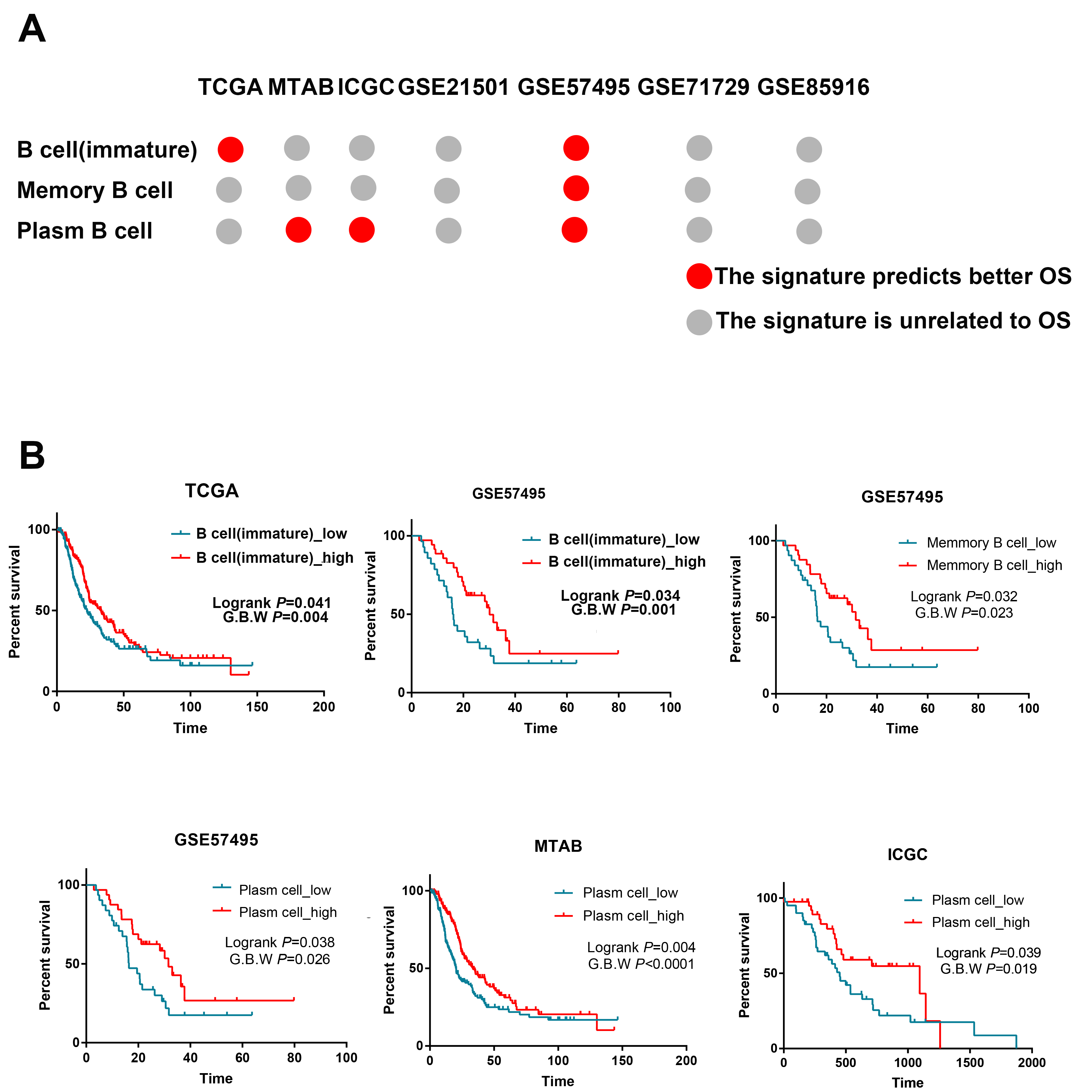

Supplement: Supplementary Figure 3 — The association between B cell signatures and the OS of PDAC patients. (A) The whole landscape. (B) The survival curve showed results with statistical significance. [file Image_3.JPEG]

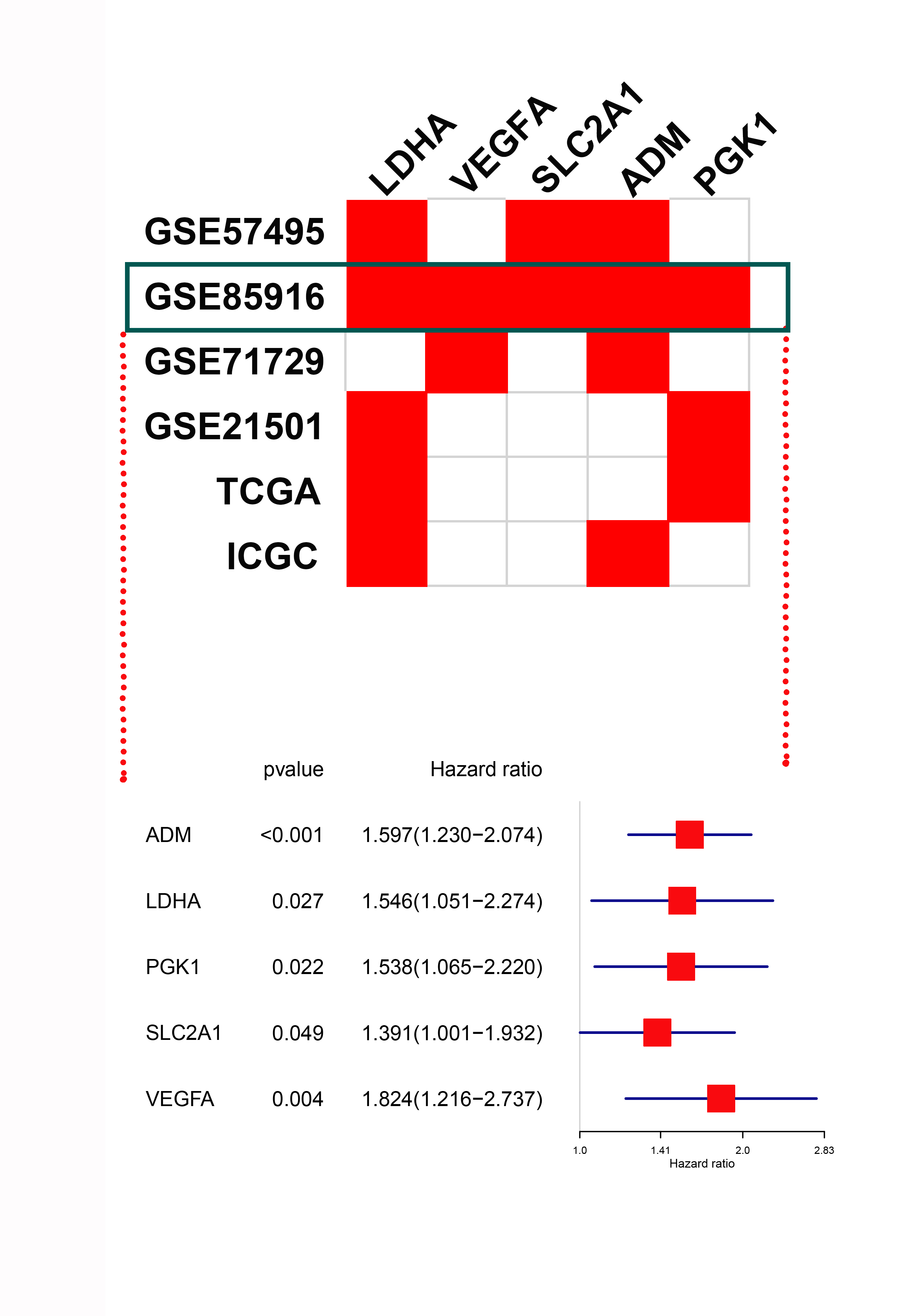

Supplement: Supplementary Figure 4 — Validation of the prognostic implications of the hub genes identified from WGCNA. [file Image_4.JPEG]

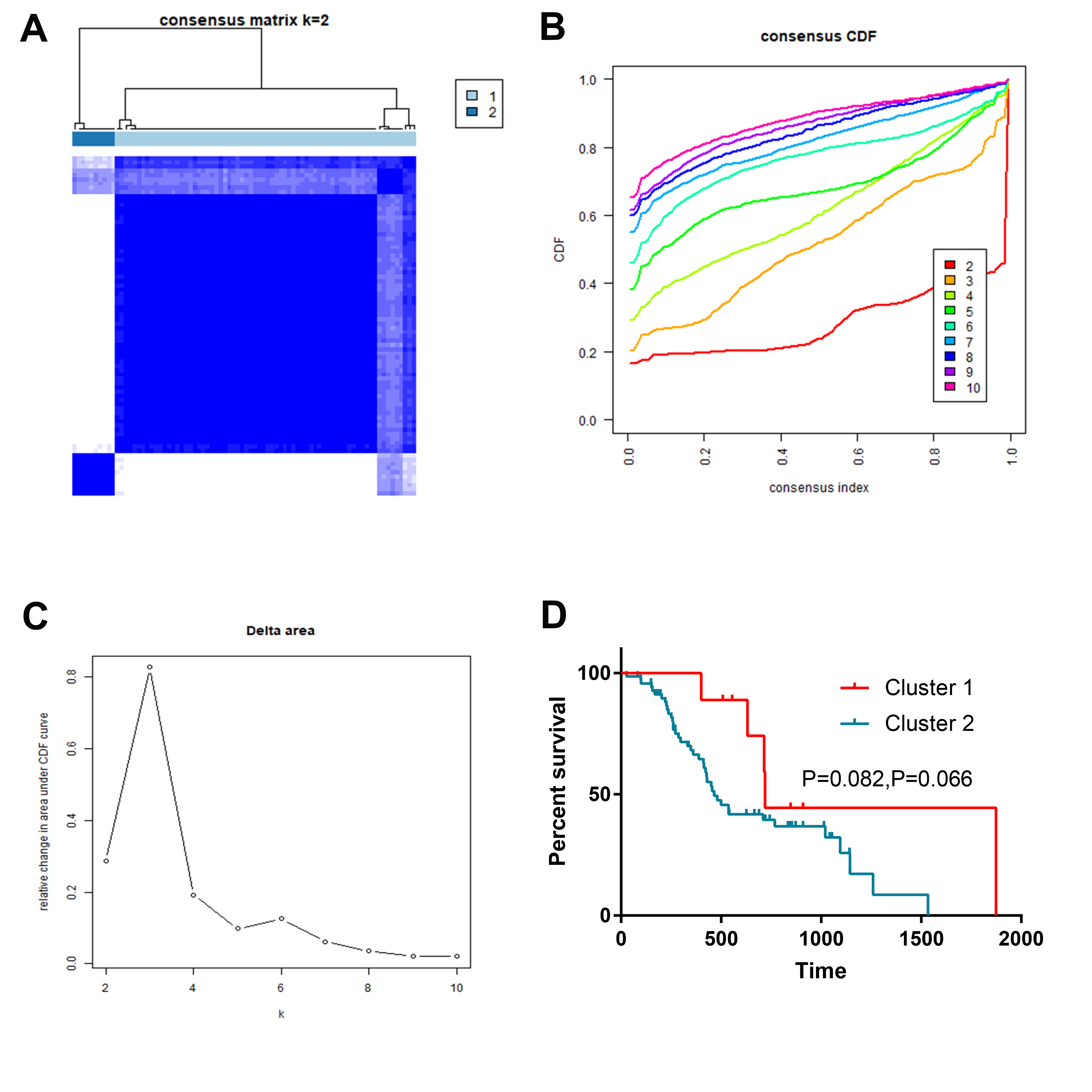

Supplement: Supplementary Figure 5 — Validation of the consensus clustering results in another dataset (ICGC). (A–C) Unsupervised consensus clustering identified two independent subclusters based on the expression levels of the differentially expressed genes between two cell states spanning the longest pseudotime. (D) Survival analysis showed that the prognosis of patients in subcluster 1 was marginally better than that of patients in subcluster 2. [file Image_5.jpg]
